# Regional heterogeneity in muscle fiber strain: the role of fiber architecture

**DOI:** 10.3389/fphys.2014.00303

**Published:** 2014-08-12

**Authors:** E. Azizi, Amber R. Deslauriers

**Affiliations:** Department of Ecology and Evolutionary Biology, University of California, IrvineIrvine, CA, USA

**Keywords:** strain differences, muscle architecture, pinnate, fusiform muscles, muscle bulging

## Abstract

The force, mechanical work and power produced by muscle fibers are profoundly affected by the length changes they undergo during a contraction. These length changes are in turn affected by the spatial orientation of muscle fibers within a muscle (fiber architecture). Therefore any heterogeneity in fiber architecture within a single muscle has the potential to cause spatial variation in fiber strain. Here we examine how the architectural variation within a pennate muscle and within a fusiform muscle can result in regional fiber strain heterogeneity. We combine simple geometric models with empirical measures of fiber strain to better understand the effect of architecture on fiber strain heterogeneity. We show that variation in pennation angle throughout a muscle can result in differences in fiber strain with higher strains being observed at lower angles of pennation. We also show that in fusiform muscles, the outer/superficial fibers of the muscle experience lower strains than central fibers. These results show that regional variation in mechanical output of muscle fibers can arise solely from architectural features of the muscle without the presence of any spatial variation in motor recruitment.

## Introduction

The mechanical output of a muscle is strongly affected by the length changes and shortening velocity of muscle fibers. The well-described force-length and force-velocity properties of muscles have long been considered important constraints on muscle performance (Hill, 1938; Gordon et al., 1966). These familiar relationships define how the force, work and mechanical power output of a muscle are affected by the length of muscle fibers as they shorten. Given this profound influence on the mechanical output of a muscle, it has been hypothesized that muscles are often restricted to operating at lengths where overlap between actin and myosin result in high forces (Lutz and Rome, 1994; Burkholder and Lieber, 2001). Muscles that are used for actions requiring high mechanical power are thought to operate at velocities corresponding to about 20–40% of maximum shortening velocity where power output can be maximized (Rome et al., 1988; Medler, 2002). In contrast muscles used primarily as force producers can be used most economically if they remain isometric, thereby maximizing force output (Roberts et al., 1997). Therefore the length changes muscles undergo can often determine their function and effectiveness during movement.

Understanding the relationship between the mechanical output of a muscle and the length trajectories of the muscle fibers has been complicated by the presence of spatial heterogeneity within muscles. The strain experienced along a muscle fascicle has been shown to vary along its length (Pappas et al., 2002; Ahn et al., 2003). Modeling approaches have shown that such variation results from the curvature of a given fiber within a muscle as well as regional variation in the mechanical properties of surrounding tissues (Blemker et al., 2005). Variation in lengths and lengths changes at the level of muscle fibers and fascicles may therefore result in significant spatial variation in muscle force.

A convenient assumption in both modeling and experimental approaches has been that strain experienced by a subset of muscle fibers is representative of the whole muscle. However this assumption is weakened by a number of complicating factors. For example regional recruitment within a muscle can result in a spatial variation in force (English, 1984) and potentially complicated mechanical interactions between active and passive muscle regions (Maas et al., 2001). In some muscles there are clear boundaries between neuromuscular compartments, which consist of different fiber types and are differentially recruited (Sokoloff et al., 1996; Carrasco et al., 1999). In some cases compartments within a muscle can perform distinct mechanical functions (Higham et al., 2008) and are associated with different motion at the level of a joint (Carrasco et al., 1999).

Even in the absence of differential motor recruitment, the architectural features of a muscle can cause regional variation in fiber strain. It is well-established that the spatial orientation of muscle fibers and fascicles within a muscle can influence the relationship between the length changes of a fiber and that of a whole muscle (Brainerd and Azizi, 2005). This is best illustrated in pennate muscles where fibers are oriented at an angle relative to the muscle's line of action. In these muscles the absolute shortening of a muscle fiber is lower than that of the whole muscle (Azizi et al., 2008; Randhawa et al., 2013; Azizi and Roberts, 2014). The amplification of fiber strain arises from the fact that fibers in a pennate muscle not only shorten along their length, they also change orientation (pennation angle) during the contraction (Otten, 1988). Given the established relationship between pennation and fiber strain, any spatial variation in architecture within a muscle can result in regional differences in fiber strain. The influence of muscle architecture on fiber strain is not limited to pennate muscles. Muscles with broad insertions can face large variation in strain if one edge of a muscle acts with a significantly different moment arm relative to the joint's center of rotation. As a result, fibers with smaller moment arms are likely to undergo relatively smaller excursions while fibers further form the joint are likely to undergo relatively large excursions (Herring et al., 1979; Van Der Helm and Veenbaas, 1991; Dean et al., 2007). Variation in fiber strain may also affect muscles with relatively simple architecture. In fusiform muscles where fibers are oriented parallel to the muscle's line of action there is likely to be little affect of fiber architecture on strain patterns. However, in these muscles the inner fibers have very low curvature compared to the fibers at the outer surface of the muscle. This variation in curvature is thought to cause differences in fiber strain between the inner and outer fibers of a fusiform muscle (Daggfeldt, 2006). Taken together, empirical and modeling results suggest that variation in fiber architecture can result in significant variation in fiber strain which in turn can influence regional contribution to force, work and power.

In this paper we combine simple geometric models of a pennate and fusiform muscle to predict the effect of fiber architecture on regional strain patterns. We tailor the models to two muscles from which we have measured regional strain patterns. We compare model and empirical results and map the degree of variation in fiber strain resulting solely from regional variation in fiber architecture.

## Pennate muscles

**Figure 1:**
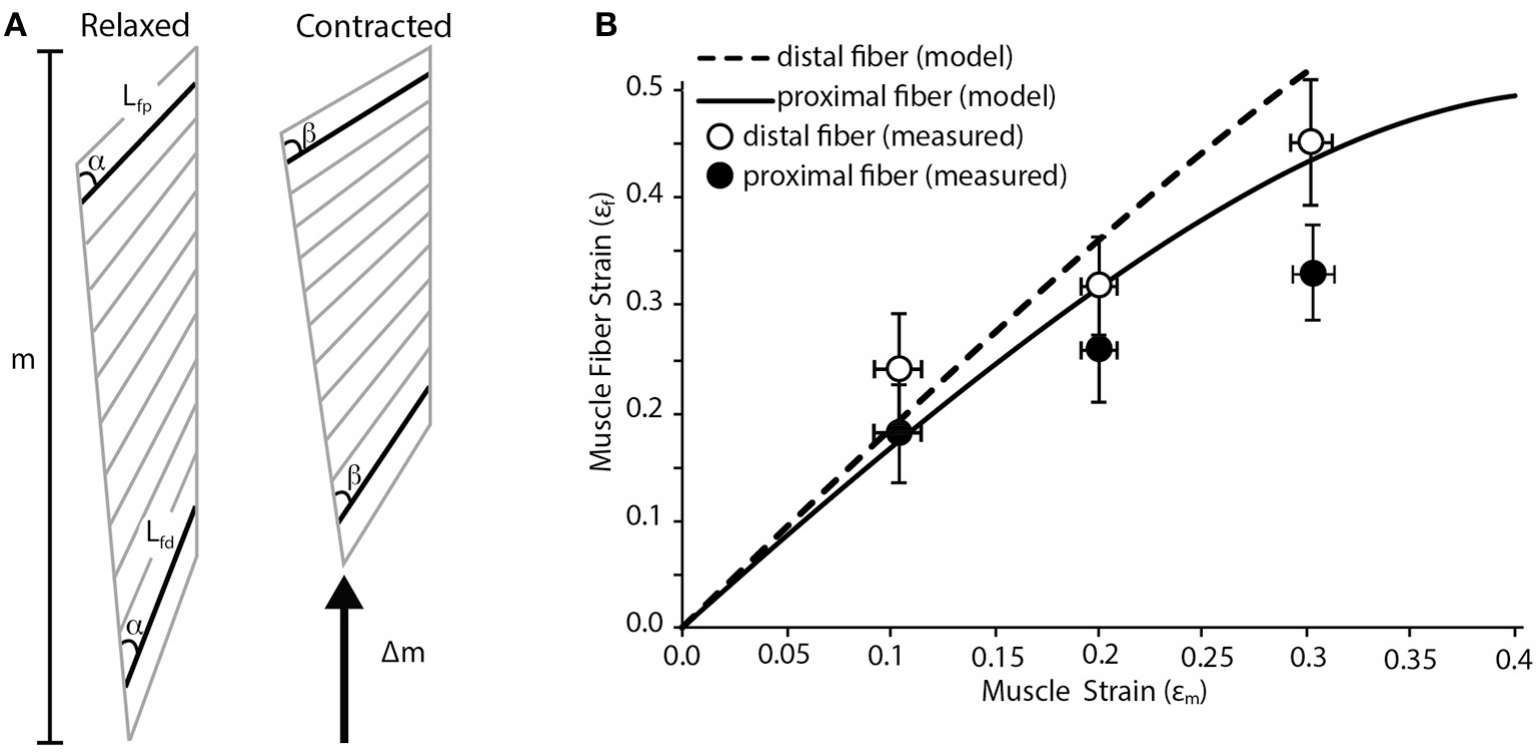
**Fiber strain heterogeneity in a pennate muscle. (A)** Schematic of pennate muscle fiber arrangement during relaxed and contracted conditions. The schematic is based on the anatomy of turkey gastrocnemius muscle. m denotes the relaxed length of the muscle. During contraction there is a change in muscle length (Δm) along the line of action of the muscle. *L*_fp_ is the length of the proximal fiber, which has a pennation angle of about 30°. *L*_fd_ is the length of the distal fiber, which has a pennation angle of about 20°. Although the initial thickness of the muscle varies along the proximal to distal axis, the thickness does not change dynamically during the contraction. There is a change in the pennation angle from angle α to β during the contraction. This change in pennation angle alters the relationship between shortening of the fibers and the shortening of the whole muscle. **(B)** A simple geometric model predicts variation in proximal and distal muscle fiber strain as whole muscle strain increases. The model predicts that higher fiber strains are associated with muscle fibers with lower angles of pennation. Closed and open circles are empirically measured mean fiber strain values from the proximal and distal fibers in the turkey lateral gastrocnemius muscle (n = 5). Error bars represent the standard error of the mean.

We use a planar model of a pennate muscle to predict regional strain patterns during a contraction. The model is based in part on work published by Benninghoff and Rollhäuser (1952). The model assumes that shortening of the fiber is coupled with changes in pennation angle (Figure 1A). This creates opposing movement in the two aponeuroses that define the insertion sites of muscle fibers. One simplifying assumption made here (and in many other pennate models) is that the thickness of the muscle (i.e., the distance between the aponeuroses) does not change during the contraction. We realize that changes in the thickness of pennate muscles have the potential to change the relationship between fiber shortening and muscle shortening (Azizi et al., 2008; Azizi and Roberts, 2013). We also assume that all fibers within the muscle are recruited simultaneously. Inputs to the model include the initial lengths of the fibers (L_f_), the pennation angle of the fibers (α) and the length change of the muscle (Δm; Figure 1A). These inputs are used to solve for the fiber strain (ε_f_) in two regions of the muscle using the following equation (Benninghoff and Rollhäuser, 1952):

(1) $$\varepsilon_{\text{f}}=1-{\left({\left(\cos\alpha -\Delta {\text{m/L}}_{\text{f}}\right)}^{2}+\sin^{2}\alpha \right)}^{0.5}$$

The dimensions inputted to the model are based on the lateral gastrocnemius muscle of wild turkeys (*Meleagris gallopavo*). In this muscle, the proximal fibers have the largest pennation angles in the muscle and pennation angle decreases distally (Figure 1A). The pennation angle of the proximal fiber is about 30° and decreases to about 20° distally. These two regions were used to estimate variation in fiber strain within the muscle.

The predictions of the simple geometric model were compared to empirically measured fiber strains in these two regions of the muscle. The methods associated with our measurements have been previously described (Roberts and Azizi, 2010). Birds were anaesthetized using inhaled isofluorane and the lateral gastrocnemius and the sciatic nerve were exposed. A custom-made nerve cuff consisting of bipolar silver electrodes was placed on the nerve. Two pairs of sonomicrometry transducers were implanted with one pair measuring the length of the proximal fascicles and a second pair measuring the length of distal fascicles. The distal tendon was severed and attached to dual-lever servomotor. The nerve was stimulated at increasing voltage until twitch force no longer increased. The maximum isometric force of the muscle was then determined during a tetanic contraction. A series of isotonic contractions were then performed where the muscle shortened against a load corresponding to 50% of its maximum isometric force. During these contractions the muscle shortened at a constant velocity. By having the muscle contract against a constant load we remove the potentially confounding effect of stretching series elastic elements. Shortening of the fibers was measured with sonomicrometry transducers while the shortening of the whole muscle was measured through the displacement of the servomotor. These measurements were then compared to the predictions of the simple geometric model.

## Fusiform muscle

**Figure 2:**
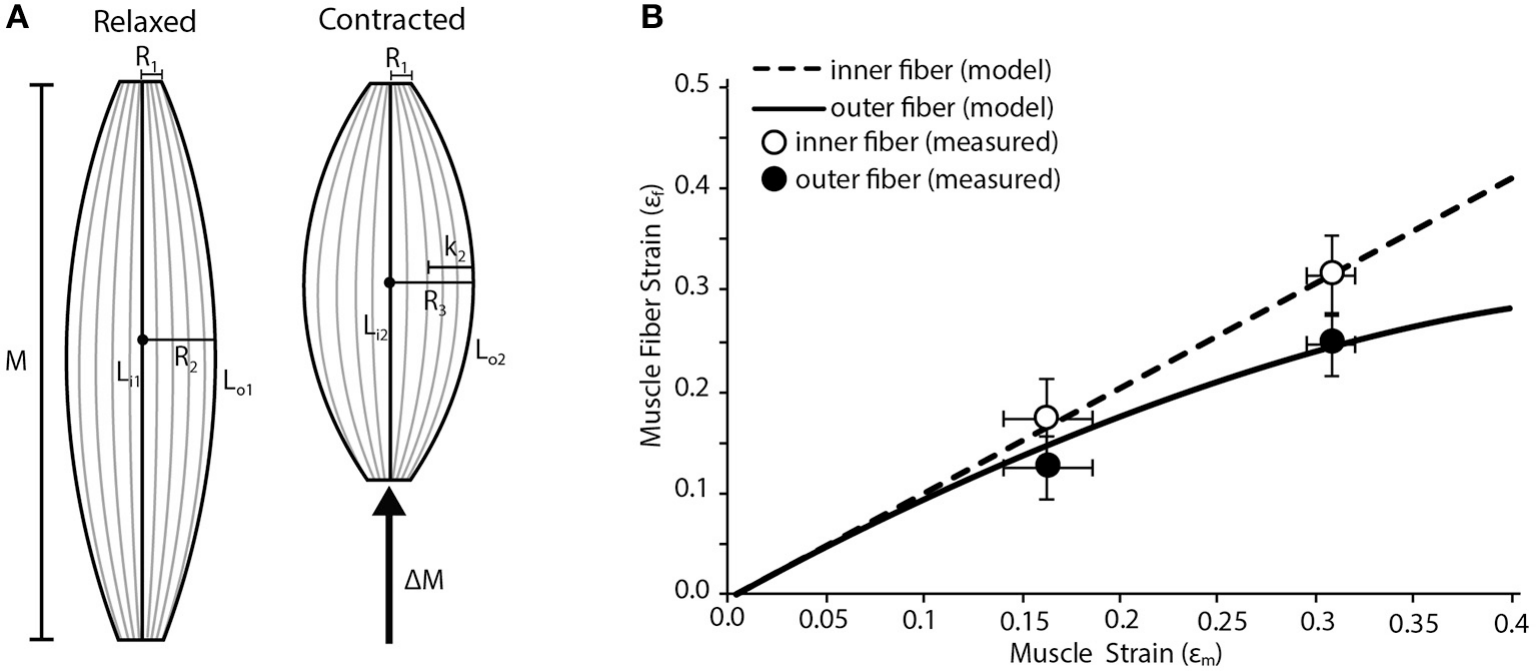
**Fiber strain heterogeneity in a fusiform muscle. (A)** Schematic of a fusiform muscle during relaxed and contracted conditions. Schematic is representative of a frog palmaris longus muscle. M denotes relaxed length of the muscle. *L*_I1_, the length of the inner fiber, is compared to *L*_o1_, the length of the outer fiber. During contraction there is a change in muscle length (ΔM) along the line of action of the muscle. *L*_I2_, the length of the inner fiber after contraction, is compared to *L*_02_, the length of the outer fiber after contraction. The radius of the muscle increases (by length *K*_2_) from *R*_2_ to *R*_3_ during contraction. *R*_1_ is the radius of the muscle where it interacts with the tendon at both the origin and insertion and is assumed to remain constant during contraction. **(B)** The model predicts variation in the strain of the innermost and outermost muscle fibers as whole muscle strain increases. The inner fibers undergo relatively higher strains than the outer fibers. Closed and open circles represent empirically measured mean strain in the outermost and innermost fibers, respectively. Data are collected from the frog palmaris longus muscle (*n* = 4). Error bars represent the standard error of the mean.

We used a simple geometric model to map fiber strains in various regions of a fusiform muscle. We model the muscle as an isovolumetric barrel (Otten, 1988). As the muscle shortens it must expand radially to maintain a constant volume. This radial expansion will increase the curvature of the outer fibers of the muscle. We predict that the, most superficial fibers of the muscle are disproportionately affected by the muscle's radial expansion creating the basis for variation in strain. We assume that the radius of the proximal and distal tendon (*R*_1_; Figure 2A) remain constant during the contraction. We also assume that the internal central fibers will undergo the same strain as the whole muscle. We input into the model the radii at the myotendinous junctions (*R*_1_), the maximum radius (midbelly) prior to contraction (*R*_2_), the initial lengths of the muscle (M), the initial lengths of inner (L_i1_) and outer (L_o1_) fibers, and the muscle strain (ε_m_). We first use these inputs to solve for the maximum radius (midbelly) at the end of the contraction (*R*_3_) using the following equation:

(2) $$R_{3}={\left(((1/1-\varepsilon_{m}))\cdot (2R_{1}^{2}+R_{2}^{2})-2R_{1}^{2}\right)}^{0.5}$$

We then solve the geometry of the muscle at the end of the contraction and solve for the final length of the fiber (L_o2_) using the following equations:

(3) $$L_{o2}=\frac{2\arctan(2k_{2}/M(1-\varepsilon_{m}))\cdot M(1-\varepsilon_{m})}{\sin(2\arctan(2k_{2}/M(1-\varepsilon_{m})))}$$

(4) $$k_{2}=R_{3}-R_{1}$$

Finally we normalize the change in fiber length to strain using the following equation:

(5) $$\varepsilon_{fo}=(L_{o1}-L_{o2})/L_{o1}$$

The dimensions inputted to the model were based on the palmaris longus muscle of leopard frogs (*Rana Pipiens*). This muscle has a fusiform shape and lacks any internal tendinous inscriptions. The shape of the muscle is ideal given some of the geometric assumptions made in the model.

The results of the model are compared to empirical measurements of the regional fiber strains in the Palmaris longus. To estimate the strains during a contraction we used an isolated muscle preparation similar to those previously described (Azizi and Roberts, 2010). The frog was euthanized with a double pithing protocol. The muscle was isolated from surrounding tissue and placed in an aerated amphibian Ringer's solution. One end of the muscle attached to a clamp controlled with a three-axis micromanipulator and the other end was attached to a dual-lever servomotor. The muscle was maximally stimulated using a pair of platinum stimulating plates. The maximum isometric force of the muscle was then determined during a tetanic contraction. A series of isotonic contractions were then performed where the muscle shortened against a load corresponding to 50% of its maximum isometric force. During these contractions the muscle shortened at a constant velocity. By having the muscle contract against a constant load we remove the potentially confounding effect of in-series compliance. The muscle was imaged from above using a ccd camera mounted onto a dissecting microscope. Muscle fiber strains were measured form the video of the contracting muscle using Image J software. The two lengths that were quantified were the outer edge of the muscle, representing the length of the outer fiber and a straight-line distance between the insertion and attachment, representing the inner fiber.

## Results and discussion

Our simple pennate muscle model predicts that the fibers with a lower pennation angle will undergo larger strains for a given amount of muscle shortening. Since the model is based on the architectural properties of the lateral gastrocnemius of wild turkeys, this suggests fibers from the distal part of the muscle will see greater strains (Figure 1B). This regional difference in fiber strain increases with increasing muscle strain resulting in about a 25% difference in fiber strain when the muscle shortens by 30%. The general trend predicted by our simple geometric model was supported by our empirical measurements of regional fiber strain (Figure 1B). We consistently found more fiber shortening in the distal fiber of the muscle with a lower pennation angle. Based on a Two-Way ANOVA we found that fiber strain increases significantly with muscle strain (*p* < 0.001) and that regional differences in fiber strain followed the predicted trend but were not statistically significant (*p* = 0.071).

In many cases, the simple geometric model predicted larger strains than those actually observed. The most likely cause of this discrepancy is the explicit assumption in the model that the thickness of the muscle remains constant during the contraction. It has been previously shown that the thickness of a pennate muscle does increase during a contraction (Azizi et al., 2008). This increase in thickness can decrease the amount shortening observed in muscle fibers (Azizi et al., 2008; Azizi and Roberts, 2013). Therefore we speculate that the incorporation of these dimensional changes during a contraction may provide better correlation between modeled and empirical measures.

Our simple fusiform muscle model predicts that fibers in the superficial or outer region of the muscle are likely to undergo lower strains than those in the central or inner region during a contraction (Figure 2B). This regional variation in fiber strain is largely due to the fact that outer fibers are disproportionately affected by lateral bulging of the muscle. Since the muscle is considered isovolumetric, any shortening along the muscle's line of action must be accommodated by the radial expansion of the muscle. This radial expansion increases the curvature of the outer fibers, which can in part resist fiber shortening. In fact, we explored some extreme conditions with our model and found that given certain starting conditions, the outer fibers could in fact be stretched when the muscle shortens substantially. Such limits are reached when the muscle starts with a high degree of curvature in the outer fibers and the muscle resembles a short and stout barrel. This suggests given certain initial muscle geometries, regional variation in fiber strain may alter the amount of regional mechanical work being performed. It may be that a fusiform muscle reaches an upper limit in mechanical work when the positive work being done by the internal fibers is completely counteracted by the negative work being done by the outer fibers (due to being actively stretched).

Our empirical measures of fiber strain in a fusiform muscle generally support the predictions of the simple model (Figure 2B). We consistently observe less fiber shortening in the outer fibers of the muscle compared to the inner fibers (Figure 2B Based on a Two-Way ANOVA we found that fiber strain increases significantly with muscle strain (*p* < 0.001) and that regional differences in fiber strain followed the predicted trend but were not statistically significant (*p* = 0.089).

An implicit assumption made in both our model and our empirical measurements is that the strain measured along fiber is representative of the strain at the level of sarcomeres. Here we are assuming that on average sarcomere lengths are homogeneous throughout the muscle. However, it is possible that alterations in the number of sarcomeres in series in fibers from different regions can function to counteract the effects of muscle architecture. For example, if the inner region of fusiform muscle has more sarcomeres in series the larger fiber strain observed would be distributed over more sarcomeres thereby reducing any effects on force production. We believe that the model and empirical data presented here can serve to inform hypothesis about regional variation in sarcomere length and number.

The empirical data presented in this study were all performed at loads corresponding to 50% of maximum isometric force. The use of isotonic contractions allowed us to make measurements of fiber length without dynamic changes in length of series elastic elements. However, previous studies have shown that the relationship between fiber strain and muscle strain can vary as a function of force (Azizi et al., 2008). Similarly, it has been suggested that the stiffness of sheet-like tendons (aponeuroses) can vary dynamically and may therefore alter the length trajectory of muscle fibers (Azizi and Roberts, 2009). Given the complexities and the force dependent features of muscle architecture it is possible that the absolute relationships between fiber strain and muscle strain may be specific to the force level selected.

The goal of this paper has been to highlight the potential for regional variation in strain and work within a muscle. Such variation may arise from purely architectural variation or could arise from regionally specific motor control strategies and well-defined neuromuscular compartmentalization (Higham and Biewener, 2011). Distinguishing between neural and architectural mechanisms can be challenging particularly in studies aimed at understanding functional heterogeneity *in vivo*. There are however, ways to quantify the contribution of fiber architecture. Animal studies have the benefit of being able to conduct muscle level experiments post-mortem. In such cases investigators can simply quantify regional fiber strain variation under maximal stimulation (similar to the present study) where all the motor units of the muscle are recruited. Any regional variation in fiber strain can be reasonably attributed to regional variation in architecture. In studies where isolated muscle experiments are not possible or practical, investigators can track regional fiber strain while passively actuating the joint or joints of interest. Again, any regional variation in fiber strain can be reasonably attributed to regional variation in architecture since the muscle is not active. Unraveling the relative contributions of structural and neural drivers of regional heterogeneity within a muscle will be an important future step in understanding muscle function during movement.

### Conflict of interest statement

The authors declare that the research was conducted in the absence of any commercial or financial relationships that could be construed as a potential conflict of interest.
